# A challenging hypothesis: can a SNP changing cysteine 1 in the α inhibin pro-domain induce reproduction-linked PCOS phenotypes?

**DOI:** 10.3389/fendo.2026.1909939

**Published:** 2026-09-23

**Authors:** Michel Roger Blanc

**Affiliations:** Laboratoire de Neuroendocrinologie Sexuelle, Institut National de la Recherche pour l'Agriculture, l'Alimentation et l'Environnement (INRA) Centre Val de Loire, Nouzilly, France

**Keywords:** alpha inhibin gene, LHRH-Statin, GnRH-Statin, PMOS/PCOS, Pro-alpha C inhibin-related molecules, SNP

## Abstract

PCOS, the leading cause of female infertility, affects 4 to 21% of women in age to give birth. Despite numerous investigations aimed at understanding the syndrome, its origin(s) remains unknown. This paper reports neglected or overlooked studies and seeks to integrate them within the relevant literature. Data presented herein strongly suggests that a modified Pro-αC inhibin (featuring a deletion of the 15 N-terminal amino acids of αC domain: “Pro-▵1-15-αC inhibin”) suppresses GnRH pulses (“GnRH-Statin activity”) *in vivo* and GnRH secretion *in vitro*. Pro-αC inhibin, an α inhibin gene product devoid of βA/βB inhibin domains and lacking inhibin or activin activity is present in follicular fluid and serum and is strongly expressed in luteal cells. A SNP, changing cysteine at position 1 to serine in the α inhibin gene Pro-domain was identified in a human polycystic ovary, in contrast to human placenta and a human macrophage cell line. This mutation which disrupts the S-S bridge linking the Pro and αC inhibin domains of Pro-▵1-15-αC inhibin or Pro-αC inhibin, is hypothesized to induce a loss-of-function. The hypothesis proposed is the following: such a loss-of-function could induce an over-activity of GnRH neurons, that has been shown to induce PCOS-like reproductive traits in female mice. Overall, if experimentally verified, this hypothesis could constitute a breakthrough in understanding the long sought after etiology (or ethiologies) for a subset of reproductive PCOS phenotypes. Furthermore, beyond the SNP hypothesis, the paper revisits Pro-αC inhibin-related molecules and their activities.

## 1 Introduction

PCOS, first described in 1935 by Stein and Leventhal (1) is a complex endocrine disorder involving both genetical, epigenetical and environmental aspects reviewed in (2). Diagnostic criteria have been defined through successive consensus conferences: US National Institutes of Health (NIH) criteria (requiring two criteria: high levels of androgens and menstrual irregularity) (3); the Rotterdam criteria established by European Society for Human Reproduction and Embryology (ESHRE) and the American Society for Reproductive Medicine (ASRM) (requiring two of the three: hyperandrogenism, menstrual irregularity, polycystic ovarian morphology (4), and the Androgen Excess and PCOS Society criteria (criteria associating -at least partly- hyperandrogenemia, hirsutism, oligo-anovulation, polycystic ovaries (5). This evolution highlights the complexity of the syndrome and criteria reviewed in (6). Consequently, prevalence estimates vary significantly based on criteria used (4-6.6% mean value for NIH parameters) -which means tens of millions of women over the world-, or according to Rotterdam criteria and for different populations or countries (4-21%) (6). Over the past decades, research efforts have intensified, as evidenced by the volume of literature: a historical survey identified 28,000 entries for “polycystic ovary” or “polycystic ovarian” between 1950 and 2015, with 20,000 of those appearing after 2001 (7). Taking advantage of the high incidence of genetic effects, polymorphism studies of candidates have been carried out (reviews (8–10): as well as transgenerational epigenetic process studies (11). Endocrine disruptors have also been suspected to be implicated reviewed in (12).

Clinical comparisons between PCOS and non-PCOS women by clinicians have revealed increased levels of i) pulsatility of plasma LH which strongly suggests high pulsatility of GnRH (13); ii) plasma LH as a consequence of increased pulsatility of LH (14); iii) plasma androgens -causing hyperandrogenism- (14); iv) plasma antimüllerian hormone (AMH or MIS, for Müllerian Inhibiting Substance) (14). These cardinal features have led to the development of numerous animal models reviewed in (15), including nonhuman primate models (16). Most of them use adult females whose mothers have been prenatally or early postnatally treated by either androgens (15) or AMH (17, 18), which induces an increase of circulating androgens. Despite these extensive efforts, the underlying causes of the syndrome remain unclear.

Although not considered by NIH or Rotterdam criteria, various features have been observed by clinicians such as insulin resistance, metabolic disease, cardiac, dermatological or psychological disfunctions (19). These features may deeply impact patient’s daily life (20). These multiple elements and the inaccuracy of the term “polycystic ovary” drove the change for the name of the syndrome now coined Polyendocrine Metabolic Ovarian Syndrome (PMOS (21),. However, the present paper will be exclusively focused on NIH and Rotterdam parameters, i.e. disregarding non-reproduction-linked disfunctions.

The present paper revisits overlooked studies, connects them with data on reproduction-linked PCOS phenotypes and proposes a compelling new hypothesis. Finally, beyond the SNP hypothesis, the paper revisits Pro-αC inhibin-related molecules and their activities. However, it does not present any new experimental data.

Almost five decades ago, a protein factor from rete testis fluid was identified that suppresses LH pulses (22, 23) and GnRH pulses (24). The term “LHRH Statin” was proposed for this activity (24) -hereafter referred to as GnRH-Statin. This activity was subsequently immunopurified using antibodies against αC inhibin peptides (25). The likely carrier of this activity is a form of Pro-αC inhibin featuring a N-terminal truncation of the αC domain (26). The hypothesis proposed here addresses the potential consequences Pro-αC inhibin loss-of-function on the reproductive phenotypes of PCOS. The discussion integrates these data with current literature, outlines experimental approaches to test this hypothesis, and analyzes the potential role of GnRH-Statin in mammalian reproductive function.

## 2 Evidence for GnRH-Statin activity

### 2.1 *In vivo* evidence

Peripheral administration of ovine *rete testis* fluid (RTF), the female counterpart of follicular fluid (FF (27), suppresses LH pulses (22, 23) and GnRH pulses (24). This effect is not mediated by an action on the LH response to GnRH. Consequently, it was concluded that RTF proteins induced a suppression of GnRH pulse frequency. Hence, the term of “GnRH-Statin” was coined to describe this new hormonal activity (24).

Third ventricle administration of RTF proteins suppresses plasma LH pulses (24). Interestingly, the active dose given by the third ventricle route is 2000 times less than that given by the peripheral route (22, 23). By contrast, plasma FSH levels were not altered after RTF administration (24), conversely to peripheral administration (22, 23).

RTF protein fractions were purified by immunoaffinity chromatography using anti N terminus peptides of αC Inhibin (25, 26). This was based on the results of Culler and Negro-Vilar (28), who observed an increase of LH pulsatility in diestrus rats shortly after passive immunization against the 1–32 N terminal peptide of αC inhibin subunit. This suggests that a blood-born molecule immunologically related to αC inhibin spontaneously decreases LH pulse frequency. The activity of the fractions was measured via third ventricle administration, monitoring effects on LH pulsatility on conscious animals. The isolated (26–28) kDa candidate (25, 26) is identical to Pro-αC inhibin (29) but features a deletion/absence of 15 amino-acid N-terminus of the αC moiety (“Pro-▵1-15-αC inhibin”). It also includes a proline at position 4 in the Pro-moiety instead of leucine identified in the bovine sequence (30), in absence of ovine Pro-inhibin moiety sequence data (31). However, it cannot be excluded that the loss of the 15 amino acid-N terminus is a consequence of purification process. The active ventricular dose is approximately 1pMole per animal (25).

Given the immunoaffinity chromatography used, the partially purified fractions likely include inhibin. Thus, it was crucial to assess if recombinant 32kDa inhibin was active in this test. However, no GnRH-Statin activity was observed using human recombinant 32kDa inhibin (32). As a consequence of a direct effect on GnRH neurons, an *in vitro* bioassay was investigated.

### 2.2 *In vitro* evidence: suppression of the GnRH release induced by GABA A receptor mediated-activation in GT1–7 neuronal cell line

The GT1–7 neuronal cell line obtained by genetically targeted tumorigenesis (33) retains certain traits of native GnRH neurons. The positive response to GABA stimulation through GABA^A^-mediated receptors observed in a fraction of native rat GnRH neurons (49-94% (34, 35) and in GT1–7 cells (36, 37) indicates that this cell line behaves similarly to a subset of native GnRH neurons.

Muscimol-induced GnRH release was totally suppressed in a dose-dependent manner by purified RTF fractions consistent with their *in vivo* GnRH-Statin activity. The effect was considered as specific particularly as no effect was observed with recombinant 32 kDa recombinant human inhibin and TGF-β(38).

Altogether, these data strongly suggests that a molecule closely related to Pro-αC inhibin, “Pro-▵1-15-αC inhibin”, present among RTF proteins carries an activity that suppresses GnRH pulses and, consequently, LH plasma pulses and LH mean levels. This likely involves a direct action on GnRH neurons (“GnRH-Statin” activity). The active dose is in the pMolar range both *in vivo* and *in vitro*.

## 3 Pro-αC inhibin molecule

### 3.1 Physico-chemical properties

Now defined by their capacity to suppress FSH secretion in pituitary cell cultures, inhibins αβA et αβB purified from FF, are heterodimers involved in reproduction and non-reproduction functions reviewed in (39). The α, βA and βB subunits constitutive of these inhibins are derived from three independent genes (39). These inhibins belong to the 42 member transforming growth factors-beta (TGF-β) superfamily including bone morphogenic proteins, growth and differentiation factors, activins, inhibins, anti-Müllerian hormone, Nodal and leftys (40). Unique to this family, the α subunit prohormone is post-translationally processed at two RXXRR/proconvertases cleavage sites, which cleaves “pro” and “αN” and “αN” and “αC” domains. From FF, besides αβA et αβB inhibins, Pro-αC inhibin molecule, was isolated and partially characterized (41), fully characterized (29, 42) and functionally studied (43). Although investigated under different names, these entities share the exact same molecular identity. Indeed, Pro-αC inhibin was also named “inhibin alpha-subunit monomer” (29), “monomeric inhibin a subunit” (41), “free inhibin a subunit” (43). Furthermore, the use of “Pro” suffix -which typically implies a non-mature form, whereas this is not the case here- has led to some confusion.

A cysteine at position 1 of Pro domain inferred in the ovine species (26) is also reported in all available cDNA sequences [Human (44, 45); porcine (45, 46); rat (47–49); mouse (50) bovine (30); equine (51); *Trichosurus vulpecula* marsupial (52); chicken (53); turkey (54); rainbow trout (55)]. No information is available concerning ovine Pro moiety sequence (GenBank accession number L28815), as this work (31) was aimed at αC inhibin domain sequencing. Thus, cysteine 1, the unique cysteine of Pro moiety (29, 44–55), is systematically conserved across these species. A S-S bridge links the Pro domain cysteine (26, 29, 42, 43), to cysteine 95 of the αC inhibin domain (56). The αC inhibin domain structure is conserved within the superfamily of TGF-βs and constitutes a compact framework (“TGFβ knot” (56),. The arrangement of the seven cysteines within the αC inhibin domain is highly conserved in eutherian mammals, reptiles, avians and fishes (40). This could suggest that Pro-αC inhibin molecule also exists in these species. Moreover, an eventual SNP changing Cysteine 1 of Pro moiety (see III) is suspected to affect similarly Pro-αC inhibin and Pro▵1-15-αC inhibin. Additionally, the αC domain of 32kDa inhibins, identical to that of Pro-αC inhibin, binds betaglycan (57, 58), however, it is not known if Pro-αC inhibin binds betaglycan. Finally, Pro-αC inhibin which is a product of the sole α inhibin gene does not exhibit any inhibin-like or activin-like activity in rat pituitary cells (29, 42). The physiological activities of Pro-αC inhibin related molecules are sumarized in **Table 1**.

**Table 1:** Pro-αC inhibin-related molecules and their GnRH-Statin, inhibin and activin activities.

Activities	GnRH-Statin	Inhibin	Activin
Pro-▵1-15-αC inhibin	Yes(26)	ND	ND
Pro-αC inhibin	ND	No(29, 42)	No(29, 42)
32 kDa inhibin	No(32)	Yes	No

ND, not determined. In parentheses are the number(s) of the reference(s).

### 3.2 Tissue expression (ovary, adrenal, other tissues)

Pro-αC inhibin, long considered to be expressed by granulosa cells as inhibins (39), is expressed principally by theca cells (43). It is involved in the activity of two genes (CYP 17A1 and INSL3) essential to androgen synthesis reviewed in (59) for CYP 17A1, and (60): for INSL3). Indeed, siRNAi-mediated knockdown of the α inhibin gene in cultured theca cells drastically depresses both CYP17A1 and INSL3 expression levels and androstenedione production (43). Importantly, the stimulating effect of LH on androstenedione production remains independent of the presence of Pro-αC inhibin (43).

The adrenal cortex cells, biologically similar to ovarian theca cells reviewed in (61), may express Pro-αC inhibin. Alpha inhibin gene is expressed in the adrenal of rats (62), sheep (63), baboon (64), and human (65–68), reviewed in (69). This expression is more abundant in the human fetus than in adult (65). A high level of expression of the α subunit compared with that of the βA or βB was observed in rats (62), sheep (63), and human (65). Alpha inhibin immunoactivity have been detected in the zona reticularis of human adrenals (65) and in human adrenal cell culture medium (70). However, the secreted peptide(s) remains to be fully identified. Thus, it is currently not known if Pro-αC inhibin is present in the adrenal or secreted by adrenal cells.

Using S1 nuclease analysis, Meunier et al. (62) identified α inhibin subunit expression not only in the rat gonads but also in the placenta, pituitary, adrenal, spleen, kidney, spinal cord and brain. The intensity of the brain signal is comparable to that of the mature testis. Like the gonads, these tissues express the α inhibin subunit at higher levels than the βA and/or βB subunits, suggesting that Pro-α C inhibin may be synthesized in these locations. Fujimura et al. (71) confirmed the α inhibin mRNA expression in various structures of the rat brain and identified the expressing cells as astrocytes based on their morphology and colocalization using both antibodies against an αC inhibin N-terminal peptide and Glial Fibrillary Acidic Protein (GFAP). However, further studies are required to identify α inhibin-like proteins secreted by these tissues.

### 3.3 Presence in biological fluids

An ELISA was designed to discriminate Pro-αC inhibin from precursors of αβA/αβB inhibins that include the Pro domain; however it does not fully discriminate between Pro-αC and “Pro-αC containing forms” (72). This fact can induce some misinterpretations of the results. A fully specific assay for Pro-αC inhibin is still necessary. However, there is little doubt that Pro-αC inhibin is present in human plasma or serum (72). Plasma concentration of Pro-αC inhibin immunoactivity has been determined during the menstrual cycle of normal women: the highest concentration is observed during mid-luteal phase. This is consistent with the observation that Pro-αC inhibin is secreted by luteal cells (43).

In human (72) and bovine FF (42), Pro-αC inhibin concentrations are equal or higher than those of αβA and αβB inhibins. This Pro-αC inhibin molecule has also likely been identified in the ovine utero-ovarian and jugular vein plasma extracts (41).

## 4 The cysteine 1 SNP hypothesis

The proposed hypothesis lies on two elements. The first element is the definitive identification of GnRH-Statin activity as being carried by Pro-▵1-15-αC inhibin. Current data (Sections I.A and I.B) strongly suggest this link, but confirmation is required. A definitive answer could be obtained using recombinant molecule. This approach would also clarify whether both Pro-▵1-15-αC inhibin and Pro-αC inhibin possess GnRH-Statin activity or not (see “DISCUSSION Testing the hypothesis” section.

The second element is based on a single-case observation. A cysteine at position 1 of the α inhibin Pro domain was identified in a human macrophage cell line (ATCC CRL 1539) (44), and human placental tissue (45). Conversely, Mason et al. (73) identified a serine 1 at this same position in an ovarian sample from a patient with PCOS. This mutation is predicted to disrupt the disulfide bridge stabilizing Pro-αC inhibin heterodimer, potentially inducing a loss-of-function. If this Cysteine 1 substitution results in a loss-of-function, the consequent disruption of the inhibitory control (GnRH-Statin activity) over GnRH neurons could lead to their upregulated activity. Such overactivity was recently shown to be sufficient to evoke hyperandrogenism, ovarian disfunction and altered estrus cycles in female mice (74), traits that are also observed in PCOS patients. In other words, the hypothesis posits that the cysteine 1 mutation contributes to, or potentially drives, certain reproduction-related phenotypes of PCOS.

However, as previously indicated, this study does not analyze other PCOS-associated traits that fall outside the diagnostic criteria of the NIH, Rotterdam, or Androgen Excess and PCOS Society Group, but which are integral part of PMOS criteria.

## 5 Discussion

### 5.1 Evidence of Pro-▵1-15-αC inhibin as GnRH Statin

This activity was purified by immunoaffinity from ovine *rete testis* fluid using antibodies directed against the N-terminal peptides of αC-inhibin moiety. The resulting fractions were then evaluated for their ability to reduce plasma LH concentrations following administration into the third ventricle of conscious rams (25). The likely carrier of this activity is Pro-αC inhibin lacking the 15 N-terminal amino acids of αC domain (Pro-▵1-15-αC inhibin).

This molecule, isolated from RTF, is a truncated form of Pro-αC inhibin purified from FF (26). The difference between the two forms could originate from their distinct sources, or alternatively, the deletion could be an artifact of the purification process. Expressing both the intact and truncated recombinant molecules to evaluate their activities *in vivo* or *in vitro* (in GT1–7 cells) would definitively resolve this question (see Section “Testing the hypothesis”). No similar work was reported in literature.

### 5.2 The Cysteine-1 SNP: a rare but potentially critical variant

Through intra-familial and twin studies, a genetic component of PCOS has been estimated to 24-72% (75, 76), respectively), which justifies the search for susceptibility locus. Given the variety of phenotypes associated with PCOS, Genome Wide Association Studies (GWAS) are considered an appropriate approach, particularly when a large number of cases and controls are required (75, 76). Extensive collaborative research efforts have been undertaken using GWAS (77–80). The most comprehensive studies to date have incorporated 10 million genetic markers across more than 10,000 probands and 100,000 controls (10, 80). Overall, these GWAS identified 11 to 16 susceptibility associated loci; however, none included the T→A SNP (cysteine-to-serine) of Pro α inhibin (localized on 2q33-35). This may be a consequence of the stringent probability cut-off employed (P = 1 x 10–^8^ to 5 x 10^-8^), which prevent false positive but simultaneously suppresses rare true-positive variants. Furthermore, it should be noted that genetic analyses comparing PCOS/PMOS probands and healthy controls have not identified this variant (81). This is in line with the lack of identification by GWAS (10, 77–80). These results suggest that T→A SNP is restricted to a single family or a limited number of PCOS cases. Nevertheless, functional data gathered in the present paper (Section 1.A and 1.B), justify a genetic analysis specifically targeted at the SNP identified by Mason et al. (73).

This work thus highlights, for the first time, a potential association between the SNP at position 1 of the α inhibin gene and a subset of reproductive PCOS phenotypes. This association was uncovered through a close reading of the first three articles that sequenced this gene (44, 45, 73), including their “Materials and Methods” sections. Furthermore, this association was made possible by previous experimental work (22–26, 32, 38).

The retrospective review of these observations could provide a useful framework for studying other multifactorial diseases in which rare but functionally critical variants can be difficult to identify.

### 5.3 Reconciling contradictory results

Central administration of crude bovine FF -which contains high amounts of Pro-αC inhibin (41–43)- was shown not to alter mean LH plasma levels in either castrated rams or ovariectomized ewes (32). In contrast, the central administration of Pro-▵1-15-αC inhibin-enriched fractions suppresses LH plasma levels (25).

First, it should be noted that the two molecules are not identical and only Pro-▵1-15-αC inhibin is currently suspected to carry GnRH-Statin activity. Utilizing recombinant forms of each molecule will provide critical insight.

Second, these contradictions could be explained if crude FF contains a molecule that counteracts GnRH-Statin activity. Although this hypothesis must be considered with caution, it should be emphasized that inhibins and activins -which act as mutual antagonists reviewed in (39)- were originally purified from FF, which, in its unpurified state, exhibits overall FSH-suppressive activity.

Antimüllerian Hormone (AMH) represents a potential candidate to such counteracting activity. Indeed, AMH is present in follicular fluid (82, 83) and was recently shown to increase LH pulsatility when administered at a 50nM dose into the lateral ventricle of mice (84). This action is mediated by AMHRII receptors identified on GnRH neurons, or at least a subpopulation of them (84). Conversely, the effective dose of Pro-▵1-15-αC inhibin observed *in vivo* is in the pMolar range (25). Nevertheless, this 50,000-fold difference concentration is substantial. First, it must be stressed that no AMH dose-response was examined in this test on LH pulsatility (84), leaving open the possibility that a much lower dose could still be effective. Second, the injection site could modulate the respective response by altering the proportion of GnRH neurons targeted. Regardless, this discrepancy challenges the AMH counteractivity hypothesis.

These elements do not preclude the existence of another, yet-uncovered counteracting activity present in FF capable of upregulating GnRH pulsatility. Such a mechanism could counterbalance inhibitory factors in women without PCOS, thereby preventing the hyperactivity of the GnRH neurons identified in women with PCOS (13). Conversely, the loss-of-function induced by the T>A SNP of Pro-▵1-15-αC inhibin could exaggerate the stimulatory activity of AMH on GnRH neurons, which has been shown to induce “PCOS-like traits in female mice” (74).

### 5.4 Does Pro-▵1-15-αC inhibin decrease GABAergic input to GnRH neurons?

Confocal microscopy and electrophysiological techniques in mice have demonstrated that the activation of GABA^A^ receptors predominantly -though not exclusively- promotes GnRH neuron activity reviewed in (85), including GnRH release (86). Furthermore, in a prenatally androgen-exposed (PNA) mouse model of PCOS, GABAergic innervation of GnRH neurons was markedly increased compared to control animals; this was associated with both an increase in GnRH/LH pulse frequency and a decrease in progesterone negative feed-back (87). Regarding the former parameter, elevated cerebrospinal fluid (CSF) concentration of GABA observed in PCOS patients also suggest an enhanced GABAergic tone on GnRH neurons (88). Similar alterations regarding the latter two parameters have also been documented in PCOS patients (89). Furthermore, given that.

1. purified fractions containing Pro-▵1-15-αC inhibin completely suppress GnRH release induced by GABA^A^ receptor-mediated activation in GT1–7 cells (Section III.B), and
2. GT1–7 cell line may reflect a sub-population of GnRH neurons, the increased GABAergic drive observed in the PNA mouse model could potentially be attenuated by Pro-▵1-15 αC inhibin in this model, and, as well as in PCOS. Further studies are required to decipher the interactions between Pro-▵1-15-αC inhibin, GABA^A^ receptors and GnRH neurons.

### 5.5 How Pro-▵1-15-αC inhibin access GnRH neurons?

Information concerning Pro-αC inhibin and Pro-del1-15αC inhibin molecules remains very limited.

First, active fractions of Pro-▵1-15-αC inhibin operate within a low dose range, specifically around the picomolar level both *in vivo* and *in vitro* (Sections III.A and III.B).

Second, the two molecules have a 26–28 kDa molecular weight.

Third, the potential involvement of GABA^A^ receptors in the mechanism of action of Pro-▵1-15-αC inhibin has been demonstrated in the GT1–7 cell line (Section III.A).

In a systematic study using Affimetrix GeneChip expression analysis, GT1–7 cells were shown to express GABA^A^ receptor α3, β2, β3, ε, and π subunits (90). The absence of any γ subunit expression is consistent with previous reports (91, 92). However, this contradicts previous studies regarding the specific subunits identified, as the only common subunit expression described across all three reports is that of β3 subunit. Further studies are necessary to solve this discrepancy.

### 5.6 Access to GnRH neurons: a potential role of betaglycan?

Betaglycan is a co-factor of the TGF-β receptor complex, featuring a short intracellular domain that lacks obvious signaling motifs (93). It also binds to inhibin (57), via the αC inhibin moiety (58), which is common to Pro-αC inhibin as well as αβA et αβB inhibins. However, the binding of Pro-αC inhibin and Pro-▵1-15-αC inhibin has not yet been examined.

Interestingly, high levels of betaglycan mRNA expression have been localized within various rat brain structures, including the cells lining the third ventricle (potentially tanycytes (94), the arcuate nucleus, and the median eminence (95). Furthermore, the presence of hypophysiotropic GnRH neurons in the arcuate nucleus is well established, alongside a high density of GnRH nerve terminals and neurons in the median eminence (86). Conversely, no known role has been ascribed to inhibins αβA et αβB in these brain structures.

Altogether, these features could suggest betaglycan as one of the several potential candidates involved in Pro-αC inhibin signaling. These observations are intriguing and warrant further investigation.

### 5.7 Testing the hypothesis

Screening PCOS patients for the Mason et al. (73) variant could be implemented very rapidly, as numerous cohorts already exist and genetic tools are readily available. However, assessing the functionality of this variant will require the development of new tools.

The first step involves the expression of both recombinant Pro-αC inhibin and Pro-▵1-15-αC inhibin, which is facilitated by the availability of their DNA sequences (44, 45). The use of these recombinant proteins, combined with *in vivo* or *in vitro* bioassays (Sections III.A and III.B), will make it possible to definitively attribute or rule out GnRH-Statin activity for Pro-▵1-15-αC inhibin, and, ultimately, for Pro-αC inhibin.

These recombinant proteins will also be used to develop a specific immunoassay targeting either Pro-αC inhibin or Pro-▵1-15-αC inhibin (or both). A sandwich ELISA appears to be the most appropriate format. One of the two antibodies will be a monoclonal antibody specifically developed against the Pro-inhibin domain and selected using recombinant Pro-αC inhibin or Pro-▵1-15-αC inhibin. The second antibody will be directed against the αC inhibin moiety.

Finally, identifying the receptor represents another crucial step: as a member of the TGFβ family, does the carrier of the GnRH-Statin activity utilize a specific type II receptor? The availability of a specific receptor would enable the development of reporter assays, which are essential for measuring variant bioactivity.

At present, no experimental studies have examined the effects of Cysteine1→Serine substitution on protein folding, secretion, receptor interactions, or biological activity of Pro-αC inhibin-related molecules. Therefore, the proposed loss-of-function mechanism has not yet been experimentally demonstrated and remains hypothetical.

### 5.8 Beyond the SNP hypothesis: revisiting Pro-αC inhibin-related molecules and activities

Throughout this paper, questions have arisen concerning the molecular identity of α inhibin gene products: are the proteins identified through α inhibin gene expression and/or αC inhibin immunoactivity true αβA/αβB inhibins, or are they Pro-αC inhibin-related molecules?

The study of Pro-αC inhibin expressed in theca cells by P.G. Knight’s group (43) revealed the androgenic role of this molecule. This androgenic activity may potentially extend to other tissues. Similarly, if it is confirmed that Pro-▵1-15-αC inhibin acts through GABA^A^ receptors (Section 1.B), this opens up new avenues for exploring functions within the brain. The tools proposed here (see previous section) will facilitate these future studies.

## 6 Conclusion

This hypothesis proposes that Pro-▵1-15-αC inhibin -or Pro-αC inhibin- associated with the cysteine-1 mutation of Pro domain is a potential upstream driver for a subset of reproductive PCOS phenotypes. As a hypothesis, this must be experimentally validated. However, even if the association between the cysteine-1 mutation and a PCOS phenotype is not verified, the primary proposal -that the Pro-▵1-15-αC inhibin or eventually Pro-αC inhibin molecule carries GnRH-Statin activity- deserves to be revisited. Confirming this would introduce a “new” actor into the complex network regulating mammalian reproductive function and would renew interest in this activity and molecular entity.

## Data Availability

The original contributions presented in the study are included in the article/supplementary material, further inquiries can be directed to the corresponding author/s.
